# Larvicidal Activity of Novaluron, a Chitin Synthesis Inhibitor, Against the Housefly, *Musca domestica*

**DOI:** 10.1673/031.006.5001

**Published:** 2006-12-31

**Authors:** Huseyin Cetin, Fedai Erler, Atila Yanikoglu

**Affiliations:** 1 Department of Biology, Faculty of Arts and Science, Akdeniz University, 07058, Antalya, Turkey; 2 Department of Plant Protection, Faculty of Agriculture, Akdeniz University, 07058, Antalya, Turkey

**Keywords:** insect growth regulator, pest management

## Abstract

A chitin synthesis inhibitor, novaluron, was evaluated under laboratory conditions for its larvicidal activity against a field population of the housefly, *Musca domestica* L. (Diptera: Muscidae), by feeding and dipping methods. The concentrations used were 1, 2.5, 5, 10 and 20 mg a.i./kg in both methods. The product caused >80% larval mortality at 10 and 20 mg a.i./kg. Of the two methods, feeding was more effective for larvicidal activity at doses above 2.5 mg a.i./kg. After 72 hours, the LC50 and LC90 values were 1.66 and 8.25 mg a.i./kg, respectively, with the feeding method; and 2.72 and 17.88 mg a.i./kg, respectively, using the dipping method. The results showed that the product provided good control of housefly larvae and would greatly reduce adult emergence.

## Introduction

The housefly, *Musca domestica* L. (Diptera: Muscidae) is an important mechanical vector of several bacterial and pathogenic organisms of humans and animals (Chavasse et al. 1999; Emerson et al. 1999). Sasaki et al. (2000) reported that houseflies were shown to spread a deadly strain of *Escherichia coli* in Japan. Zurek et al. (2001) showed that adult houseflies can transmit *Yersinia pseudotuberculosis*. In some parts of the world where cholera and typhoid are common, they also transmit these diseases (WHO, 1991). *M. domestica* is the most common insect species found in homes, horse stables, poultry farms, and ranches.

Because of its importance as a public health pest, many insecticides have been used directly or indirectly in the control of *M. domestica*. Throughout the world, houseflies have developed resistance to these insecticides. Recently, 10 generations of selection of field-collected houseflies produced a strain that was highly resistant (>150-fold) to spinosad (Shono & Scott, 2003). In Denmark, Kristensen and Jespersen (2003) found resistance toward diflubenzuron, and field populations with some resistance to cyromazine. Many researchers found similar results for other synthetic and biological insecticides (Kaufman et al. 2001; Learmount et al. 2002; Marcon et al. 2003). As levels of insecticide resistance continue to increase, it is ever more important to develop alternative methods and insecticides for controlling the housefly.

Novaluron is a chitin synthesis inhibitor that acts by both ingestion and contact (Ishaaya et al. 1996, 1998). It has been used for controlling a variety of insect pests of field crops (Ishaaya et al. 2003) and of public health (Mulla et al. 2003). Su et al. (2003) reported that novaluron exhibited a high level of activity against *Culex* mosquitoes. Mulla et al. (2003) showed that novaluron exhibited long-term activity against *Aedes aegypti* in water-storage containers.

The present study was undertaken to investigate larvicidal activity of novaluron (OSCAR Super® EC-10), against *M. domestica* under laboratory conditions.

## Materials and Methods

### Chemicals

A 1% emulsion concentrate of novaluron (OSCAR Super^®^ EC-10) was obtained from Oben Ltd. Sti. Ankara, Turkey. Concentrations for the present study were determined by a series of preliminary studies that yielded between 25 and 95 percent larval mortalities.

One ml formulation was dissolved in 100 ml distilled water (stock solution) and from the stock solution different concentrations; 1, 2.5, 5, 10 and 20 mg a.i./kg were prepared.

### Housefly strain

The strain of *M. domestica* originated from adults collected on a farm in Topcular, Antalya, using a sweep net and colonized at the Biology Department, Faculty of Arts and Science, Akdeniz University. The rearing method was that of Kristensen and Jespersen (2003) with some modifications. Adults of *M. domestica* were fed milk and dry sugar. Bran and milk was prepared at a weight ratio of 1:3, and 50 g of this mixture was placed on a plastic plate as an oviposition site.

### Feeding method for the bioassay test of the larvae

Feeding with novularon was carried out according to the method described by Kristensen and Jespersen (2003) with some modifications. To test the toxicity of the product, portions of artificial larval rearing medium (one portion: 400 g wheat bran, 200 g lucerne meal, 10 g baker's yeast, 500 ml whole milk, and 500 ml water) were treated with different concentrations of novaluron. Twenty grams of medium were put in a container and 2.5 ml of water containing novaluron or water alone was added and mixed into the medium. Twenty-five newly laid housefly eggs were placed on a piece of wet black filter paper that fit into a beer bottle cap. The eggs adhered well to the filter paper, and the caps with the eggs were placed inverted on top of the media in the containers. After 24–36 hours eggs hatched and larva began to feed. The number of emerging flies was recorded 3 weeks after the egg-seeding date and the larvicidal activity was recorded as the percentage of hatched eggs that were unable to develop into adults. All larvicidal assays were carried out at 12:12 (L:D) photoperiod, 60 ± 10% RH and 26 ± 2 °C in the laboratory until emergence of all flies. Five concentrations of novaluron were used, plus a control. Four replicates of twenty-five larvae each were used for each treatment. The experiment was repeated three times on subsequent days.

### Dipping method for the bioassay test of the larvae

The dipping method was applied according to the method described by Sukontason et al. (2004) with some modifications. All tests were run at 12:12 (L:D) photoperiod, 60 ± 10% RH, and 26 ± 2 °C. First instar larvae, 0–2 hours old, were used in the assays. Four replicates of twenty-five larvae each group were used at each concentration level. The experiments were repeated on subsequent days. The larvae of each group were gently dipped into insecticide solutions with a dip net, whereas those of the controls were dipped in tap water. After being dipped for exactly 30 sec, the larvae were transferred to the rearing jars containing food. After the larvae had been dipped, they were reared to determine the success of emergence. The number of emerging flies was recorded during the 3 week-test period.

### Statistical analysis

The larval mortality data were corrected for control mortality by the formula of Abbott (1925). ANOVA was carried out with SPSS 10.0. Means were compared with Duncan's multiple range test (p<0.05) and LC50, LC90 values were calculated by using the EPA computer probit analysis program (Version 1.5).

## Results and Discussion

The larvicidal activity of novaluron against larvae of *M. domestica* is presented in Table 1. Larval mortalities increased significantly (P<0.05) with dose using both treatment methods. Concentrations of 10 and 20 mg a.i./kg resulted in over 80% larval mortality in both assays. Mortality was generally greater in those larvae treated by feeding as compared to the dipping method. Some of emerging adults showed morphological abnormalities (malformations of wing, leg, head, thorax and abdomen) at these concentrations. When larvae were fed with the concentration of 20 mg a.i./kg, they completed only the first instar, however, and died during ecdysis.

**Table 1:**
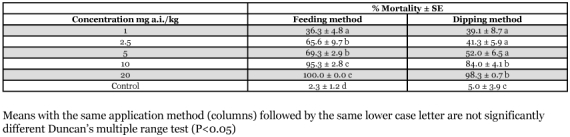
Larvicidal activity of novaluron against *Musca domestica*

From the probit analysis the LC50 and LC90 were 1.66 and 8.25 mg a.i./kg, respectively, for feeding assays; and 2.72 and 17.88 mg a.i./kg, respectively, for dipping assays.

Many different IGRs have been tested against the housefly. Webb and Wildey (1986) reported that diflubenzuron was effective in controlling strains of housefly that were resistant to carbamate, organophosphorus, organochlorine, and pyrethroid insecticides on a United Kingdom pig farm; 416 mg/m^2^ applied to slurry pots in pig weaning stalls gave effective control for 2–4 weeks after application. Howard and Wall (1995) tested triflumuron against the housefly under laboratory conditions and found that a concentration of 1 milliunits (mU)/gram of triflumuron applied topically to adult females of *M. domestica* resulted in egg hatch inhibition greater than 95%. Similarly, Wright et al. (1975) found that 0.1 and 1 mg/kg of TH 6040 incorporated in rhinoceros feed inhibited development of *M. domestica* in the feces.

The data obtained from the present study clearly indicate that novaluron could provide excellent larval control of *M. domestica*. In addition, it was found that both feeding and dipping application methods had high larvicidal activity at the higher concentrations, 10 and 20 mg a.i./kg, and both of them could be used in the control of housefly. These results suggest that novaluron may be used in housefly control programs. Further assays regarding the effect of novaluron under field conditions should be followed.
